# Color Match of Single‐Shade Versus Multi‐Shade Resin Composites: A Systematic Review With Meta‐Analysis

**DOI:** 10.1111/jerd.13444

**Published:** 2025-02-22

**Authors:** Eleonora Forabosco, Uros Josic, Ugo Consolo, Luigi Generali, Carlo D'Alessandro, Lorenzo Breschi, Vittorio Checchi

**Affiliations:** ^1^ Department of Surgery, Medicine, Dentistry and Morphological Sciences With Transplant Surgery, Oncology and Regenerative Medicine Relevance University of Modena and Reggio Emilia Modena Italy; ^2^ Department of Biomedical and Neuromotor Sciences University of Bologna Bologna Italy

**Keywords:** color match, direct restoration, meta‐analysis, multi‐shade, resin composite, single‐shade, systematic review

## Abstract

**Objective:**

To address the following PICOS question: Can single‐shade resin composites achieve a color match comparable to multi‐shade composites in tooth restoration?

**Materials and Methods:**

A comprehensive search was conducted across multiple electronic databases to identify in vitro and clinical studies evaluating the color match in tooth restoration, in terms of CIELAB (Δ*E*_*ab*_) and/or CIEDE2000 (Δ*E*_00_) color differences metrics, using single‐shade and multi‐shade composites. The risk of bias was assessed using the Quality Assessment Tool For In Vitro Studies (QUIN Tool), while the revised Cochrane Collaboration's tool (RoB 2) was employed for randomized controlled clinical trials (RCTs). Meta‐analyses were performed using RevMan to compare Δ*E*_*ab*_ and Δ*E*_00_ values between single‐shade and multi‐shade composites (*p* < 0.05).

**Results:**

After initial screening, 15 in vitro studies and 4 RCTs met the inclusion criteria for qualitative synthesis, with 8 in vitro studies selected for quantitative analysis. The majority of in vitro studies were classified as medium risk of bias, while RCTs were ranked as low risk of bias. Meta‐analyses performed on in vitro studies revealed that single‐shade composites exhibited statistically significant higher color differences with the surrounding tooth structure compared to multi‐shade composites, for both Δ*E*_*ab*_ and Δ*E*_00_ (*p* < 0.05). However, RCTs usually reported promising outcomes for single‐shade materials.

**Conclusions:**

This systematic review concluded that multi‐shade composites provide a more accurate color match between tooth and direct restoration than single‐shade materials when evaluated by instrumental analysis in laboratory settings.

**Clinical Significance:**

Multi‐shade composites exhibit superior color match properties in laboratory studies. However, single‐shade composites are a promising alternative when observed visually in clinical settings.

## 1 Introduction

The primary objective of restorative dentistry is to restore tooth function while replicating its original shape and color [1, 2]. In direct restorations, resin composites are the most commonly used materials due to their excellent mechanical and optical properties [3, 4]. To meet the increasing patients' esthetic demands [5, 6], achieving an ideal color match between natural tooth tissues and the restorative material is crucial [7], so that no difference can be detected by the human eye [8].

Color is a psycho‐physical response to the interaction of light energy with an object and the subjective experience of the observer [9]. In esthetic dentistry, the layering technique for direct composite restorations, first introduced in the 1980s [10], remains “the gold standard” for mimicking the polychromatic nature of natural teeth [11]. This technique achieves optical matching between tooth tissues and restorative materials through color layering, and the final color of a restoration results from multiple composite masses with varying chromas and opacities [10]. However, attaining perfect color harmony is challenging due to the differing thickness distributions, structures, compositions, and optical properties of enamel and dentin [12]. Thus, traditional multi‐shade composites, which vary in shades, chromas, and opacities, require advanced restorative skills, complex shade selection process, and increased cost and chair‐side time [13, 14].

In recent years, dental manufacturers have developed optically advanced materials that can reflect the color of surrounding dental tissues, facilitating good esthetic outcomes [15]. These innovative universal single‐shade composites are designed to match a wide range of classical shades, thereby reducing restorative procedure time and simplifying the color selection phase in direct restorations [5]. After placement, these composites can reflect specific tooth color wavelengths and adapt chromatically to surrounding tooth structures, providing natural shade adjustment [16]. This optical phenomenon is often referred to as the “chameleon effect” or “blending effect.” [17].

Despite the promising clinical implications, the concept of shade simplification has yielded controversial results in laboratory studies when comparing single‐shade to multi‐shade composites [7, 13, 18, 19], while few clinical trials have been published only recently [20, 21, 22, 23].

Therefore, the aim of this systematic review was to evaluate the color match capability of single‐shade composites compared to multi‐shade composites in direct restorations. Specifically, we aimed to address the following PICOS question: “Can single‐shade resin composites achieve a color match comparable to multi‐shade composites in tooth restoration?”

## 2 Materials and Methods

### 2.1 Study Protocol and Registration

The review protocol was registered in the International Prospective Register of Systematic Review (PROSPERO) database under the number CRD42024501788. The reporting of this systematic review followed the PRISMA 2020 guidelines [24].

### 2.2 Eligibility Criteria and Search Strategy

The PICOS question [25, 26] that guided the search criteria and inclusion of articles was as follows: Population (P): sound extracted human teeth with standardized cavities and/or adult (> 18 years old) dental patients in need of direct composite restorations; Intervention (I): single‐shade composite restorations placement; Comparison (C): traditional multi‐shade composite restorations placement; Outcome (O): color match (instrumental and/or visual evaluation) between surrounding tooth structure and direct restorations; Study design (S): in vitro and in vivo (randomized controlled clinical trials–RCTs) studies.

The exclusion criteria were as follows: (1) reviews (narrative or systematic); (2) case reports; (3) conference abstracts, dissertations, and theses; (4) studies on primary dentition; (5) studies on teeth with enamel defects; (6) studies that included devitalized teeth; (7) cavities made on resin teeth; (8) studies not evaluating color difference; (9) studies involving indirect restorations; (10) studies other than the English language. No date limitation was established during the literature search.

The search strategy and keywords are listed in Table S1.

### 2.3 Study Selection and Data Extraction

After completing the database search, all articles were imported into a web platform (Rayyan ISR, Cambridge, MA, USA) [27]. The last systematic literature search was conducted on November 2, 2024, while the last manual search was conducted on December 19, 2024. Titles and abstracts of each study were independently evaluated by two reviewers (E.F. and C.D.A.).

For full‐text assessment, articles that appeared to fit the inclusion criteria or lacked sufficient information in the title and abstract were chosen. In case of disagreements, a third reviewer (V.C.) was consulted.

A custom‐made spreadsheet (Microsoft Office Word 2019; Microsoft Corporation, Redmond, WA, USA) was used to extract data from the included studies. These details included the study and year of publication, the type and number of single‐shade composite restorations (test group), the type and number of multi‐shade composite restorations (control group), the aging and storage conditions for in vitro studies, the CIELAB (Δ*E*_*ab*_) and/or CIEDE2000 (Δ*E*_00_) color differences metric calculated at various time points, and results. If any data were missing or unclear, the corresponding author of the study was contacted via e‐mail to retrieve the missing information.

### 2.4 Risk of Bias Assessment

Two authors (E.F. and U.J.) independently evaluated each included paper's quality and risk of bias. The parameters were evaluated according to the Quality Assessment Tool for In Vitro Studies (QUIN Tool) [28]. This tool involves the evaluation of 12 criteria. For each article, a score was given for each criterion evaluated as adequately specified (2 points), inadequately specified (1 point), not specified (0 point), or not applicable (criteria excluded from the calculation). After the evaluation, the scores for each criterion were summarized to determine the total score for the specific in vitro study. These scores were then used to classify the study as high, medium, or low risk of bias based on the percentage obtained. A score of over 70% indicated a low risk of bias, 50%–70% indicated a medium risk of bias, and less than 50% indicated a high risk of bias.

The updated Cochrane Collaboration tool for evaluating the risk of bias in RCTs, known as RoB 2 [29], was employed for in vivo studies. The RoB 2 tool [29] features algorithms that interpret responses to specific signaling questions to propose a risk of bias judgment for each outcome assessed in a study. Hence, the evaluation criteria were categorized into five areas: (1) bias from the randomization process; (2) bias due to deviations from the intended interventions; (3) bias due to missing outcome data; (4) bias in outcome measurement; and (5) bias in the selection of reported results. The bias risk judgment for each of these five areas was classified as either “low risk of bias,” “some concerns,” or “high risk of bias.” The overall bias risk for a specific outcome in a study was determined based on the classification within these assessment domains, as recommended by the RoB 2 tool. If at least one domain was rated as “some concerns” and all other domains as “low risk,” the overall bias risk could be “some concerns.” If multiple domains were rated as “some concerns,” the overall risk of bias could either be “some concerns” or “high,” depending on the investigators assessment. Therefore, if any domain was rated as “high risk of bias,” the overall bias risk had to be considered “high.”

### 2.5 Meta‐Analysis

Meta‐analyses were performed using RevMan (Review Manager 5.4, The Cochrane Collaboration, Copenhagen, Denmark). A random effects model was used to compare the mean difference for Δ*E*_*ab*_ and Δ*E*_00_ between single‐shade and multi‐shade composites. The level of significance was set to *p* < 0.05. Heterogeneity was assessed using the Cochran *Q* test and the *I* ^2^ index.

## 3 Results

### 3.1 Study Selection

**FIGURE 1:**
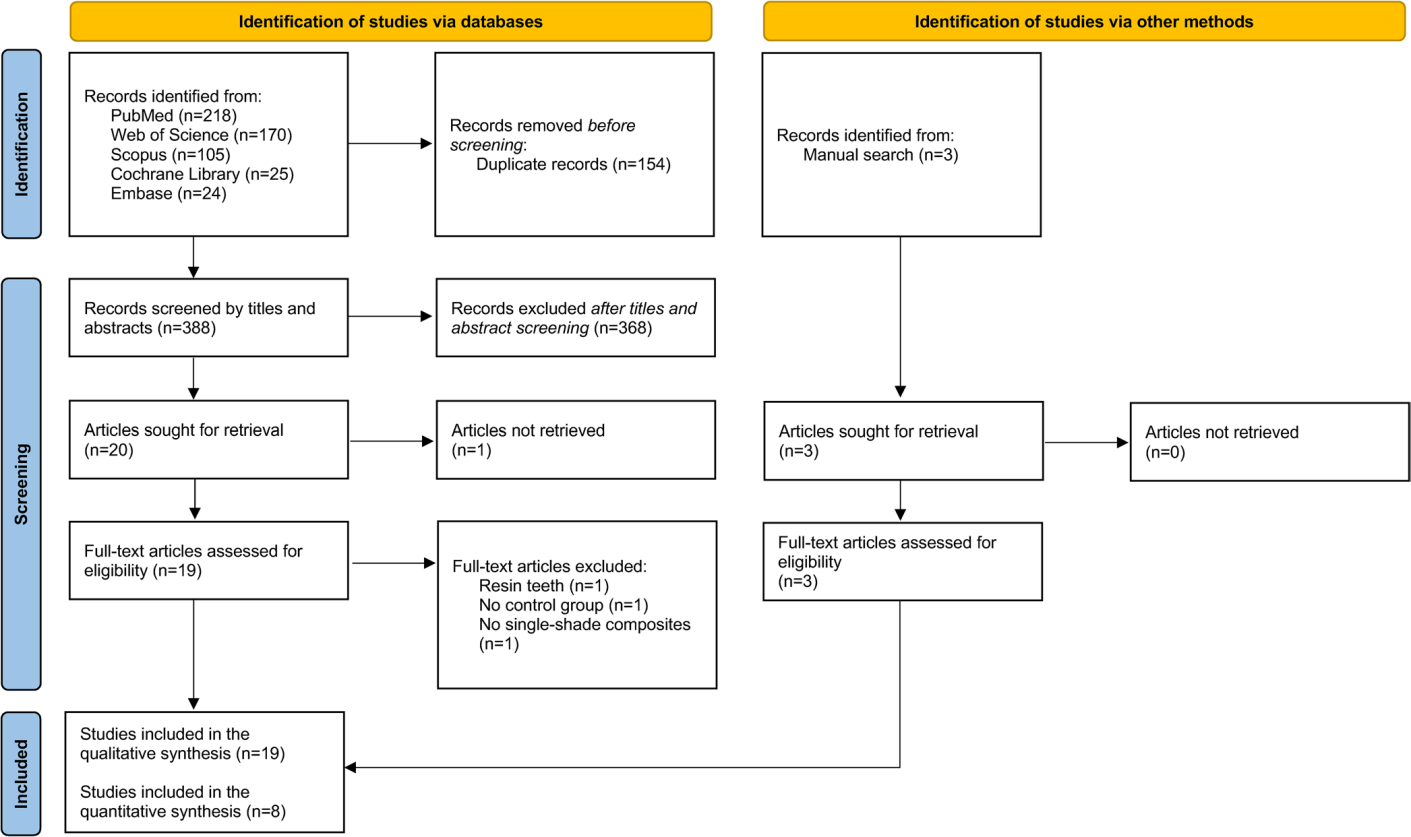
Flowchart according to the PRISMA statement.

Figure 1 presents a flowchart outlining the procedure for selecting studies in accordance with the PRISMA statement.

A total of 542 articles were retrieved from all databases. After removing duplicates, the titles and abstracts of 388 papers were screened.

Following the application of exclusion criteria, 20 studies remained for full‐text evaluation. From these, the full text of one manuscript could not be retrieved [30], and three studies were excluded because of the following reasons (Figure 1): (1) cavities made on resin teeth [13], (1) no control group [31], (1) no single‐shade composite was used [32]. Two eligible studies were identified from citation searching [33, 34].

Nineteen articles were included in the qualitative analysis: 15 in vitro studies and 4 RCTs. Seven in vitro studies could not be included in the meta‐analysis because, in six studies, the mean and SD could not be extracted [33, 35, 36, 37, 38, 39], and in 1 study only visual analysis was performed [40]. Consequently, 8 laboratory studies were included in the quantitative analysis [34, 41, 42, 43, 44, 45, 46, 47]. RCTs [20, 21, 22, 23] were not included in the meta‐analysis due to methodological heterogeneity.

### 3.2 Descriptive Analysis of the Selected Studies

Detailed information about the 19 articles [20, 21, 22, 23, 33, 34, 35, 36, 37, 38, 39, 40, 41, 42, 43, 44, 45, 46, 47] included in this review is shown in Tables S2 and S3.

All in vitro studies were conducted in university settings, with the majority of them carried out in Egypt [33, 34, 37, 44], followed by Brazil [40, 42, 47], Japan [35, 38, 39], Turkey [41, 43], Saudi Arabia [36, 45], and the USA [46]. The studies were published between 2021 and 2024. A total number of 666 extracted human anterior and posterior teeth were used across the analyzed in vitro studies: 676 cavities were reconstructed using single‐shade, while multi‐shade composites were applied to 626 cavities.

Some of the studies included “split tooth design” when evaluating the color match potential of the tested materials [36, 37, 45, 47]. The included in vitro studies evaluated the color match using the CIEL*a*b* system used to calculate color differences through Δ*E*_*ab*_ and/or Δ*E*_00_ formulas. The majority of the studies [33, 34, 36, 37, 41, 42, 44, 45, 47] used VITA Easyshade (Vita, Zahnfabrik, Bad Sackingen, Germany), three studies [35, 38, 39] used CIE XYZ digital camera (RC500, PaPaLaB, Shizuoka, Japan), 1 study [43] used Spectro ShadeTM Micro (MHT Optic Research, Milan, Italy), one study [46] used CristalEye (Olympus, Tokyo, Japan). Solely visual analysis was performed in one laboratory study [40].

The 4 RCTs were conducted in Egypt [20], Brazil [21, 23], and India [22]. A total of 316 anterior and posterior restorations (140 single‐shade and 176 multi‐shade) were placed in 161 patients older than 18 years using the split‐mouth design. As for cavity configuration, patients with at least two non‐carious cervical lesions [21, 23] or occlusal carious lesions [20, 22] were included. In 1 study [20], the depths of class I cavities were not specified; however, it was noted that cavities with “considerable depth” were excluded after carious lesion removal. In another RCT [21], most non‐carious cervical lesions had a depth between 1 and 2 mm, with only 4 cavities exceeding 2 mm. In the third RCT [22], class I cavities in the single‐shade group were restored with Omnichroma Blocker applied to the cavity floor to mask discolorations from secondary caries or old amalgam fillings. Two RCTs [20, 22] used Omnichroma as single‐shade composite, 1 study [21] used Admira Fusion X‐tra, while 1 study [23] used Vittra APS unique.

The color evaluation methods used in RCTs were as follows: in two of the RCTs [21, 23], VITA Easyshade (Vita, Zahnfabrik, Bad Sackingen, Germany) was used to calculate Δ*E*_00_ and/or Δ*E*_*ab*_; a DSLR Camera (Canon 13 D) and a digital Adobe Photoshop software both registered color coordinates to calculate Δ*E*_00_ in another RCT [22]. Finally, one RCT [20] performed solely visual analysis—which had also been performed in the above‐mentioned RCTs. Modified USPHS [20, 22] and FDI [21, 23] criteria were used across the studies.

The follow‐up period in RCTs ranged from baseline to 18 months.

### 3.3 Risk of Bias of the Included Studies

**FIGURE 2:**
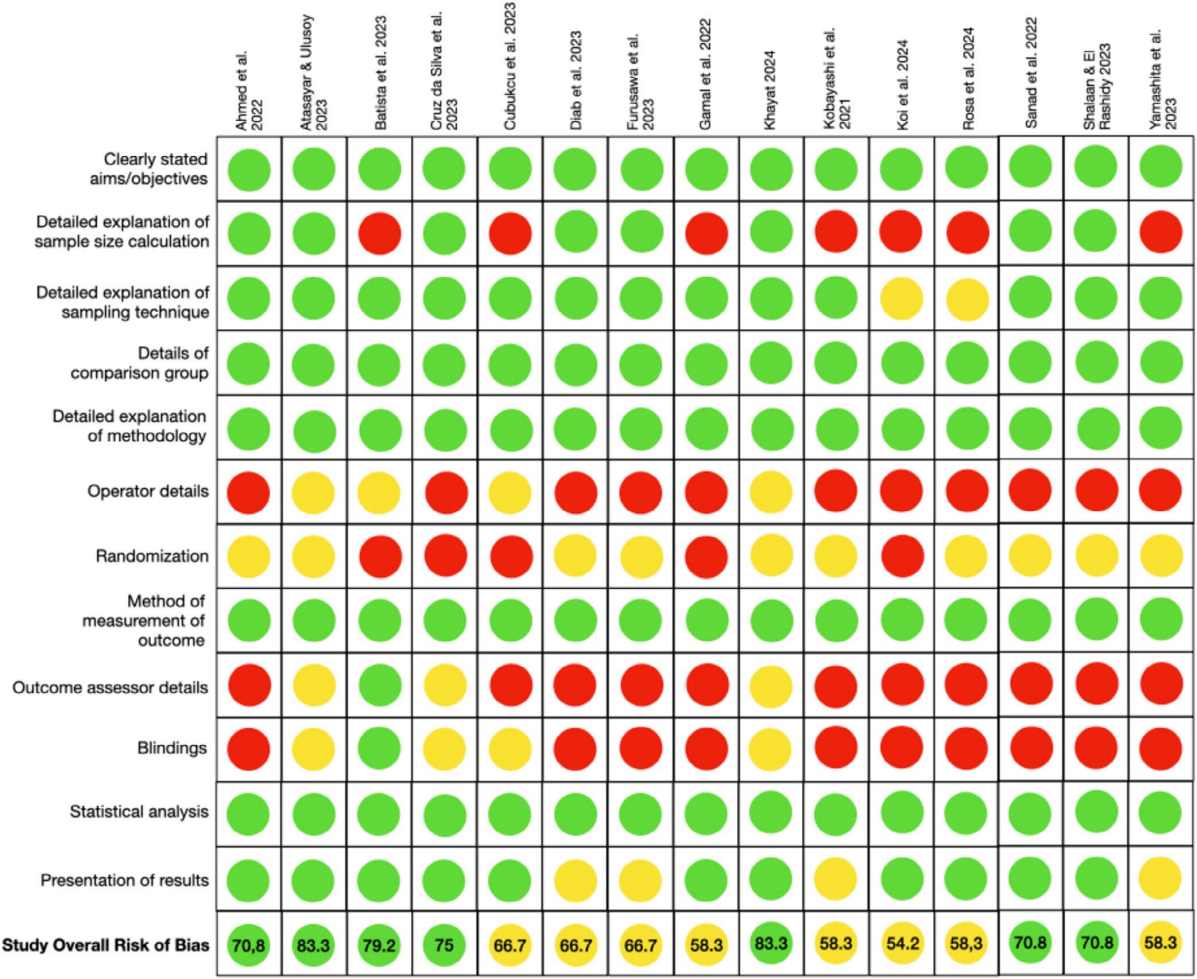
Risk of bias analysis of in vitro studies according to the QUIN tool.

**FIGURE 3:**
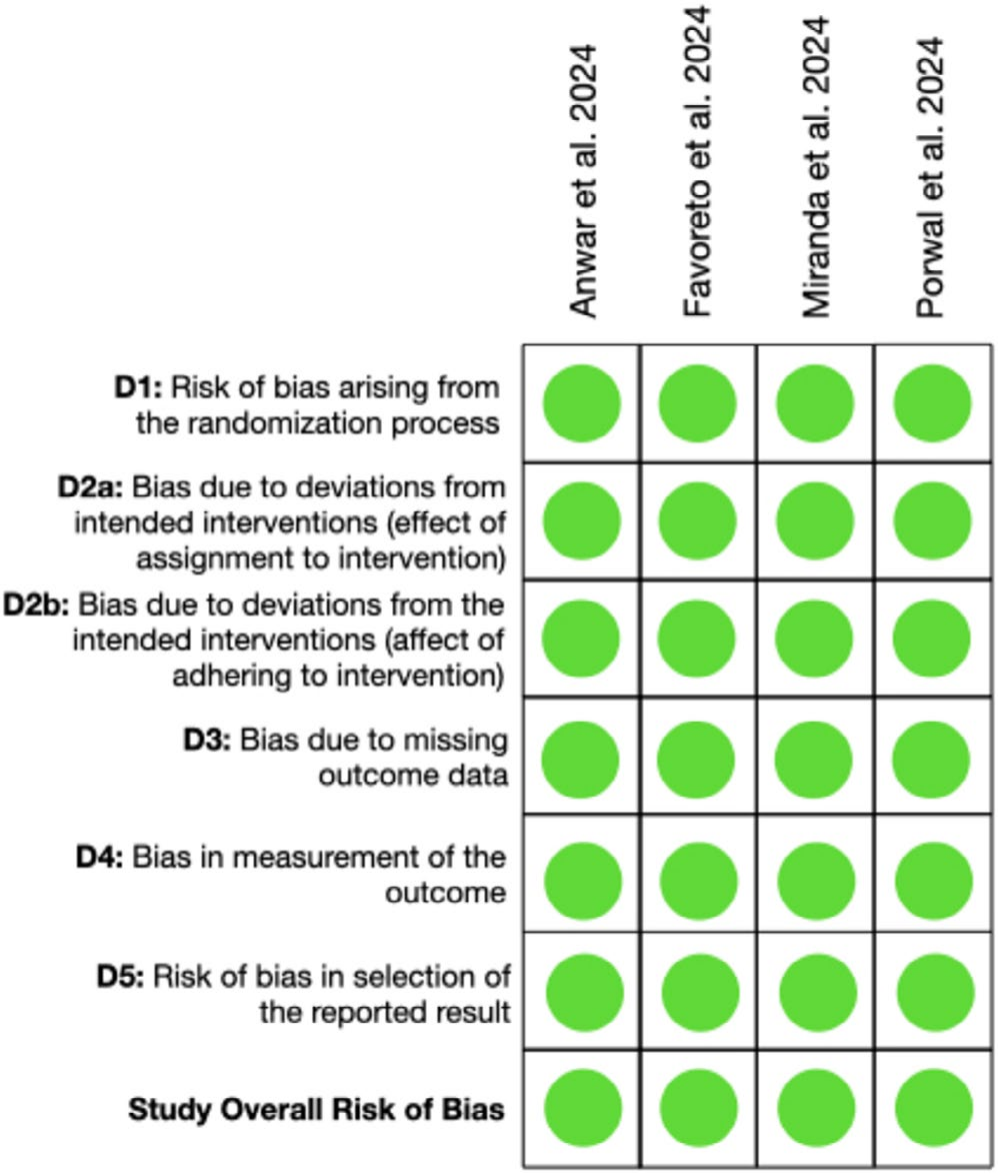
Risk of bias analysis of randomized controlled clinical trials according to the RoB 2 tool.

Figures 2 and 3 provide an overview of the risk of bias evaluation [28, 29]. The majority of in vitro studies were labeled as having a medium risk of bias [33, 35, 37, 38, 39, 43, 46, 47]. Seven studies revealed a low risk of bias [34, 36, 40, 41, 42, 44, 45], and no study showed a high risk of bias. The problematic domains included outcome assessor details [33, 34, 35, 36, 37, 38, 39, 43, 44, 46, 47], operator details [33, 34, 35, 36, 37, 38, 39, 42, 44, 46, 47], blinding [33, 34, 35, 36, 37, 38, 39, 44, 46, 47], sample size calculation [35, 37, 39, 40, 43, 46, 47], and randomization [37, 40, 42, 43, 46].

All 4 RCTs [20, 21, 22, 23] were ranked as low risk of bias (Figure 3).

### 3.4 Meta‐Analysis

**FIGURE 4:**
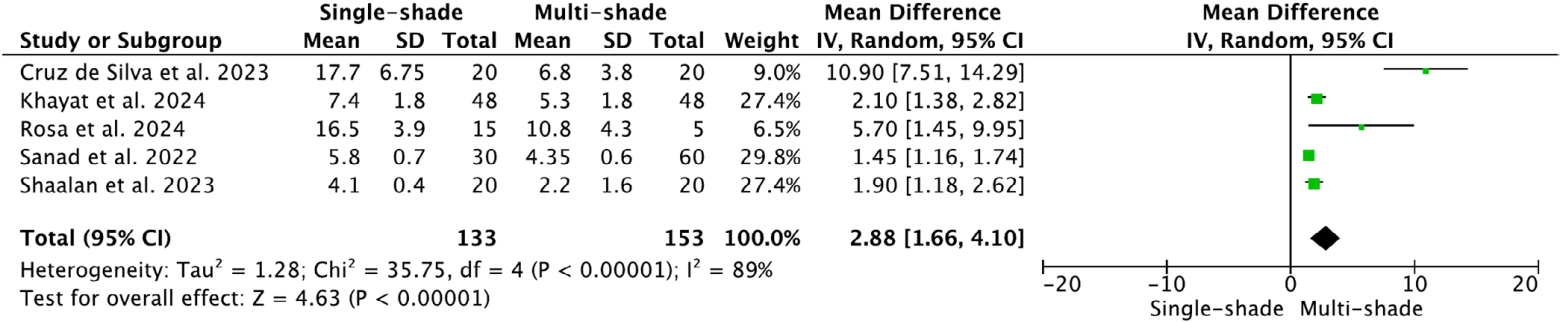
Forest plot of Δ*E*_*ab*_ of single‐shade and traditional multi‐shade composites.

**FIGURE 5:**
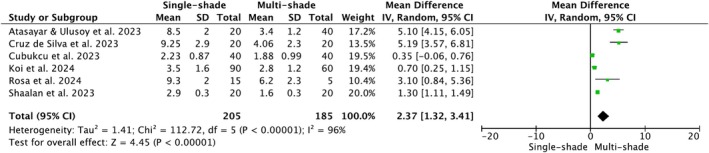
Forest plot of Δ*E*_00_ of single‐shade and traditional multi‐shade composites.

Within the first week after restorations' placement and specimens' storage in distilled water, single‐shade composites exhibited statistically significant higher color differences with the surrounding tooth compared to multi‐shade composites for both Δ*E*_*ab*_ (*p* < 0.00001; MD = 2.88, 95% CI [1.66, 4.10]) and Δ*E*_00_ (*p* < 0.00001; MD = 2.37, 95% CI [1.32, 3.41]) formulas. A high heterogeneity index among the studies was found for Δ*E*_*ab*_ (*I* ^2^ = 89%) and Δ*E*_00_ (*I* ^2^ = 96%) (Figures 4 and 5, respectively).

In the forest plot for Δ*E*_*ab*_ (Figure 4), the studies by Cruz de Silva et al. (2023) [42] and Rosa et al. (2024) [47], which significantly shift the diamond far to the right, evaluated the color‐match potential after 3 and 7 days, respectively, in contrast to other studies that conducted evaluations after 1 day. Sensitivity analysis revealed that, by excluding these two studies with different time points, the heterogeneity reduced from *I* ^2^ = 89% to 43%. Regarding the forest plot for Δ*E*_00_ (Figure 5), it was difficult to detect the source of higher heterogeneity, even after running a sensitivity analysis.

## 4 Discussion

The dental industry has made significant efforts to develop “universal” materials such as adhesive systems, resin composite cements, and resin composites. These innovative materials aim to allow greater tolerance to technique sensitivity without compromising the functional and esthetic outcomes of restorations [48, 49]. Recently, many manufacturers have introduced their own universal or single‐shade composite, but only some of these have been tested in in vitro studies. The most frequently used composite in the in vitro studies [33, 34, 35, 36, 37, 38, 39, 41, 42, 43, 44, 45, 46, 47] included in this review was Omnichroma—the first product that has been launched to the market—followed by Vittra APS Unique, Charisma Diamond One, Admira Fusion X‐tra, and Essentia Universal [40, 42, 46, 47]. While these single‐shade composites may exhibit good color adjustment due to high translucency and uniform spectral reflectance [50], our meta‐analyses indicated that traditional multi‐shade composites usually achieve a superior color match when in vitro studies are analyzed.

The extent of color change or adjustment can be evaluated visually or by using instruments [18]. Instrumental color analysis can detect VITA scale values and all CIEL*a*b* coordinates: L* (lightness from 0 = black to 100 = white), a* (chroma and hue on the red‐green axis), and b* (chroma and hue on the yellow‐blue axis) [7]. These values allow for calculating color differences between tooth and composite materials using formulas developed by the Commission Internationale de l'Eclairage (CIE) [51, 52]. The CIELab formula (Δ*E*_*ab*_), a simplified version of CIEDE2000 (Δ*E*_00_), lacks adjustments such as weighting functions (SL, SC, and SH), parametric factors (kL, kC, and kH), and the rotation function (RT) that accounts for chroma and hue interactions, particularly in the blue region [12]. Therefore, Δ*E*_00_ is more precise and computationally demanding than Δ*E*_*ab*_, designed to reduce discrepancies between calculated and perceived color differences [51, 53]. Interestingly, the results from our meta‐analyses revealed statistically significant differences for both ∆*E*_*ab*_ and ∆*E*_00_ when comparing the color match ability of single‐shade to multi‐shade composites.

Due to their translucency, single‐shade composites reflect the color of the surrounding cavity walls, implying that the cavity depth affects their blending ability. Consequently, the cavity depth and the thickness of the composite restoration influence its color [47]. A recent study [39] explained that single‐shade materials are unable to replicate the color of the surrounding tooth structure when the cavity depth reaches 2 mm, as their ability to mirror the underlying substrate is substantially reduced. In most of the studies included in our meta‐analysis, the depth of the restored cavities was 2 mm [41, 42, 43, 44, 46, 47] or 1.5 mm [34, 45]. This might explain why we found that single‐shade composites were less performant compared to multi‐shade materials when their color match was assessed by means of instrumental analysis for both Δ*E*_*ab*_ and Δ*E*_00_ formulas. Our results should, however, be interpreted cautiously due to considerable heterogeneity indices observed among the studies. Although two separate meta‐analyses were conducted for data pooled for Δ*E*_*ab*_ and Δ*E*_00_ values obtained from specimens stored in distilled water within the first week after restoration placement, the heterogeneity was high, most likely arising from variations in the materials used (e.g., different single‐shade composites utilized across the in vitro studies) and different evaluation time points [26]. Indeed, it should be mentioned that the color match potential of single‐shade composites is reported to be material (or brand) dependent [39, 54, 55]. This implies that, to achieve the optimal esthetic outcome, the material should be selected cautiously, taking into consideration also the material's brand, rather than just the category [49].

Unlike instrumental evaluations, visual assessments with a shade guide are subjective, influenced by the observer's skills, gender, age, and lighting conditions [56]. Despite these subjective influences, it remains the most commonly used procedure in clinical practice. Although the visual method may have drawbacks, it is often decisive in patient acceptance and satisfaction [42, 57]. Conversely, instrumental assessments, favored in dental research for their objective results, provide quantifiable data that can detect fine color differences not perceptible to the human eye [16].

Trained observers use visual analysis to assess the overall esthetic harmony and integration of restorations, considering factors like translucency, texture, and gloss that instruments might overlook [58]. Human vision adapts to slight color variations, often deeming minor mismatches identified by spectrophotometers as clinically acceptable, emphasizing the restoration's seamless blend with natural dentition [8, 17]. This may explain why, in some cases, visual inspection revealed similar or even better color adjustments of single‐shade composites in several reviewed in vitro studies [41, 42, 43, 47]. It is noteworthy to mention that visual analysis revealed that multi‐shade composites may achieve a better color match than single‐shade composites when the tooth color is A3.5, A4, B4, or C4 [40, 45].

The RCTs included in this systematic review had no issues regarding the risk of bias assessment, and they were all judged as low risk according to the RoB 2 tool, implying excellent randomization and blinding technique—factors that can be crucial when evaluating the color of direct restorations. In general, the authors of the analyzed RCTs reported no statistically significant differences between multi‐shade and single‐shade restorations for all follow‐ups (from baseline to 18 months) [20, 22]. In 1 RCT [22], after 9 and 12 months, multi‐shade composites displayed higher Alpha scores and a lower prevalence of Bravo scores for color match and marginal staining according to the modified USPHS when compared to single‐shade materials. Nevertheless, both Alpha and Bravo scores were considered as “clinical success” Interestingly, 1 RCT [23] with the longest follow‐up (18 months) reported excellent clinical behavior for both single‐ and multi‐shade composites and comparable color match of the two materials when evaluated both visually and instrumentally.

A recently published systematic review and meta‐analysis of RCTs [59] answered the following PICOS question: “Do single‐shade resin‐based composites have shade performance comparable to that of multi‐shade resin composites in direct restorations?” Four RCTs were included [20, 21, 60, 61], and no statistically significant differences were found between single‐shade and multi‐shade composite resins from 1 to 7 days and at 12 months when restorations were assessed by visual inspection. Although some of the RCTs were not included in our systematic review due to exclusion criteria (i.e., studies performed on primary teeth [61]), our findings generally align with those from Leal et al. (2024) [59] and confirm promising potential of single‐shade materials to achieve good color match up to 18 months of clinical service.

Lastly, some important observations deriving from this systematic review should be mentioned: in vitro studies should standardize cavity dimensions when preparing samples, improve blinding procedures, and consistently perform sample size calculations. Additionally, better randomization techniques should be employed to minimize bias. Conducting visual analyses alongside instrumental evaluations is crucial for comprehensive assessments of color matching. By addressing these aspects, future in vitro research can provide more accurate and clinically relevant implications. Finally, longer‐term studies and more RCTs should provide insights into the effects of aging on single‐shade restorations' color, particularly if it adapts to tooth color changes from dietary habits [12] over time.

## 5 Conclusions

According to the findings of this systematic review, multi‐shade resin composites can provide a better color match between tooth and direct restoration compared to single‐shade materials when an instrumental analysis is performed in laboratory settings. Nonetheless, the emerging clinical evidence suggests that single‐shade composites can be a promising alternative to multi‐shade materials in the posterior region based on visual assessment.

## Conflicts of Interest

The authors declare no conflicts of interest.

## Supporting information


**Table S1.** Electronic databases and search strategies.

**Table S2.** Characteristics of the in vitro studies that were a part of qualitative analysis.
**Table S3.** Characteristics of the clinical trials that were a part of qualitative analysis.

## Data Availability

Data sharing not applicable to this article as no datasets were generated or analysed during the current study.
