# Supplementary material for: Color Match of Single‐Shade Versus Multi‐Shade Resin Composites: A Systematic Review With Meta‐Analysis
Source: J Esthet Restor Dent. 2025 Feb 22;37(6):1443–51. doi: 10.1111/jerd.13444 (PMC12087942; doi:10.1111/jerd.13444)
Supplement: Supplementary file 1 — Table S1. Electronic databases and search strategies. [file JERD-37-1443-s001.docx]

**Supplementary Table 1:** Electronic databases and search strategies.

| **Electronic Database (number of hits)** | **Search strategy** |
| --- | --- |
| **PubMed** (n=218)  <https://pubmed.ncbi.nlm.nih.gov> | (tooth restoration OR dental restoration OR "Dental Restoration, Permanent"[Mesh] OR restorative dental material OR tooth filling OR dental filling) AND ("resin composite*" OR "composite resin*" OR "Composite Resins"[Mesh] OR "restorative material*") AND (single-shade OR single shade OR universal shade OR one-shade OR one shade OR multi-shade OR multi shade OR multiple shade) AND (color match* OR shade match* OR color adjustment potential OR color perception OR "Color Perception"[Mesh] OR "Color"[Mesh]) |
| **Web of Science** (n=170) <https://www.webofscience.com> | TS=(tooth restoration OR dental restoration OR "Dental Restoration, Permanent" OR restorative dental material OR tooth filling OR dental filling) AND TS=("resin composite*" OR "composite resin*" OR "Composite Resins" OR "restorative material*") AND TS=(single-shade OR single shade OR universal shade OR one-shade OR one shade OR multi-shade OR multi shade OR multiple shade) AND TS=(color match* OR shade match* OR color adjustment potential OR color perception OR "Color Perception" OR "Color") |
| **Scopus** (n=105)  <https://www.scopus.com> | ("tooth restoration" OR "dental restoration" OR "Dental Restoration, Permanent" OR "restorative dental material" OR "tooth filling" OR "dental filling" ) AND ("resin composite*" OR "composite resin*" OR "Composite Resins" OR "restorative material*" ) AND (single-shade OR "single shade" OR "universal shade" OR one-shade OR "one shade" OR multi-shade OR "multi shade" OR "multiple shade" ) AND ("color match*" OR "shade match*" OR "color adjustment potential" OR "color perception" OR "Color Perception" OR Color) |
| **Cochrane Library** (n=25) <https://www.cochranelibrary.com> | (tooth restoration OR dental restoration OR [mh "Dental Restoration, Permanent"] OR restorative dental material OR tooth filling OR dental filling) AND (("resin" NEXT composite*) OR ("composite" NEXT resin*) OR [mh "Composite Resins"] OR ("restorative" NEXT material*)) AND (single-shade OR single shade OR universal shade OR one-shade OR one shade OR multi-shade OR multi shade OR multiple shade) AND (("color" NEXT match*) OR ("shade" NEXT match*) OR color adjustment potential OR color perception OR [mh "Color Perception"] OR [mh Color]) |
| **Embase** (n=24)  <https://www.embase.com> | ('tooth restoration' OR 'dental restoration' OR 'dental restoration'/exp OR 'restorative dental material' OR 'tooth filling' OR 'dental filling' ) AND ('resin composite*' OR 'composite resin*' OR 'resin'/exp OR 'restorative material*' ) AND (single-shade OR 'single shade' OR 'universal shade' OR one-shade OR 'one shade' OR multi-shade OR 'multi shade' OR 'multiple shade' ) AND ('color match*' OR 'shade match*' OR 'color adjustment potential' OR 'color perception' OR 'color vision'/exp OR 'color'/exp) |
