# Supplementary material for: Color Match of Single‐Shade Versus Multi‐Shade Resin Composites: A Systematic Review With Meta‐Analysis
Source: J Esthet Restor Dent. 2025 Feb 22;37(6):1443–51. doi: 10.1111/jerd.13444 (PMC12087942; doi:10.1111/jerd.13444)
Supplement: Supplementary file 2 — Table S2. Characteristics of the in vitro studies that were a part of qualitative analysis. Table S3. Characteristics of the clinical trials that were a part of qualitative analysis. [file JERD-37-1443-s002.docx]

**Supplementary Table 2.** Characteristics of the *in vitro* studies that were a part of qualitative analysis.

| **STUDY** | **TYPE & NUMBER OF SAMPLES** | **TOOTH SHADES** | **MULTI- & GROUP SHADE COMPOSITE, NUMBER & TYPE OF RESTORATION & ΔE±DS** | **SINGLE-SHADE COMPOSITE, NUMBER & TYPE OF RESTORATION & ΔE±DS** | **COLOR MEASUREMENT DEVICE** | **VISUAL SCALE** | **STORAGE** | **FOLLOW-UP** | **MAIN RESULTS** |
| --- | --- | --- | --- | --- | --- | --- | --- | --- | --- |
| *Ahmed et al. 2022* | N=60 extracted human maxillary premolars. | A2, A3, B2 and B3 (n=15) | Filtek Z250  (3M ESPE; St. Paul, MN, USA)  n=60 V class cavities (5x3x2mm) on the palatal side | Omnichroma  (Tokuyama Dental Corporation, Tokyo, Japan)  n=60 V class cavities (5x3x2mm) on the buccal side | Vita Easyshade V Compact  (VITA, Zahnfabrik, Bad Sackingen, Germany) | N/A | N/A | T_0_: after curing | No data at T_0_. |
| *Atasayar & Ulusoy 2023* | N=120 extracted human anterior teeth | Lighter colored tooth shades (A1, B1, C1, A2, B2) (n=60)  Dark colored tooth shades (A3, A3.5, B3, B4, C3) (n=60) | Estelite Sigma Quick (Tokuyama Dental, Japan)  N=40 Round shape cavities (7x2mm) on the buccal side  n=20 Blocker:  ΔE_00_=3.5±1.7  n=20 Without blocker:  ΔE_00_=3.7±0.9  Neo Spectra ST (Dentsply Sirona, Germany)  N=40 Round shape cavities (7x2mm) on the buccal side  n=20 Blocker:  ΔE_00_=3.2±1.5  n=20 Without blocker:  ΔE_00_=3.1±1.5 | Omnichroma  (Tokuyama Dental Corporation, Tokyo, Japan)  N=40 Round shape cavities (7x2mm) on the buccal side  n=20 Blocker:  ΔE_00_=7.8±1.3  n=20 Without blocker:  ΔE_00_=8.5±2.0 | VITA Easyshade Compact  (VITA Zahnfabrik, Bäd Säckingen, Germany) | 0: complete full up/no difference  1: very good match up/small difference  2: good match up/acceptable  3: bad match up/not very acceptable  4: disharmony/ completely unacceptable | Distilled water 37°C | T_0_: 24h | Instrumental evaluation: single-shaded composites exhibited higher ∆E_00_ values compared to multi-shade and group-shade.  Visual evaluation: single-shade composites achieved the best color match followed by group-shade and multi-shade composites. |
| *Batista et al. 2023* | N=23 extracted human upper and lower molars | A2 (n=1), A3 (n=2), A3.5 (n=2), A4 (n=1), B3 (n=4), B4 (n=3), C2 (n=2), C3 (n=6), C4 (n=1), D3 (n=1) | Admira Fusion  (Voco)  N=23 I class cavities (4x4mm) | Admira Fusion x-tra  (Voco)  N=23 I class cavities (4x4mm) | N/A | FDI criteria:  Score 1: Clinically excellent/very good  1.1 Luster comparable to enamel  2a.1 No surface staining  2b.1 No marginal staining  3.1 Good color match, no difference in shade and/or translucency  Score 2: Clinically good  (after polishing probably very good)  1.2.1 Slightly dull, not noticeable from speaking distance  1.2.2 Some isolated pores  2a.2 Minor surface staining, easily removable by polishing  2b.2 Minor marginal staining, easily removable by polishing  3.2 Minor deviations in shade and/or translucency  Score 3: Clinically sufficient/satisfactory (minor shortcomings, no unacceptable effects, but not adjustable w/o damage to the tooth)  1.3.1 Dull surface but acceptable if covered with film or saliva  1.3.2 Multiple pores on more than one-third on the surface  2a.3 Moderate surface staining, not esthetically unacceptable  2b.3 Moderate marginal staining, not esthetically unacceptable  3.3 Distinct deviation but acceptable. Does not affect esthetics:  3.3.1 More opaque  3.3.2 More translucent 3.3.3 Darker  3.3.4 Brighter  Score 4: Clinically unsatisfactory (but reparable)  1.4.1 Rough surface, cannot be masked by saliva film, simple polishing is not sufficient. Further intervention necessary  1.4.2 Voids  2a.4 Unacceptable surface staining; major intervention necessary for improvement 2b.4 Pronounced marginal staining; major intervention necessary for improvement  3.4 Localized clinical deviation that can be corrected by repair  3.4.1Tooopaque  3.4.2 Too translucent  3.4.3 Too dark 3.4.4 Too bright  Score 5: Clinically poor (replacement necessary)  1.5 Very rough, unacceptable plaque retentive surface  2a.5 Severe surface and/or subsurface staining, generalized or localized, not accessible for intervention  2b.5 Deep marginal staining not accessible for intervention  3.5 Unacceptable. Replacement necessary | Purified water | T_0_: 1 week | Visual evaluation:  Nonsignificant differences between the composites were observed for all scores.  For darker shades (A4, B4, C4) most of the evaluators considered the restorations made with Admira Fusion more esthetic than with Admira Fusion x-tra, For other shades nonsignificant differences were observed. |
| *Cruz da Silva et al. 2023* | N=40 extracted human teeth upper central incisors, upper and lower molars | A1, A2, A3, A3.5, and A4 | Filtek Z250  (3M ESPE; St. Paul, MN, USA)  N=20 cylindrical cavities (4x2mm)  ΔE_ab_=6.8±3.8  ΔE_00_=4.06±2.3 | Omnichroma  (Tokuyama Dental Corporation, Tokyo, Japan)  N=10 cylindrical cavities (4x2mm)  ΔE_ab_=18.6±8.6  ΔE_00_=9.8±3.6  Vittra APS Unique (FGM, Joinville, Brazil)  N=10 cylindrical cavities (4x2mm)  ΔE_ab_=16.8±4.9  ΔE_00_=8.7±2.3 | Easyshade Advance 4.0  (VITA Zahnfabrik, Bäd Säckingen, Germany) | 1: mismatch/ completely unacceptable  2: poor match/hardly ac- ceptable  3: good/ acceptable match  4: close match/small diference  5: exact match/no color diference | Distilled water | T_0_: 72h | Instrumental evaluation: multi-shade resin presented lower values of ΔE for both CIELAB and CIEDE 2000.  Visual evaluation: single- shade composites showed better matching values than the multishade resins, although all groups demonstrated acceptable color matching. |
| *Cubukcu et al. 2023* | N=80 extracted human incisors | A3 | Clearfil Majesty Esthetic (Kuraray Noritake, Tokyo, Japan)  N=40 cavities (3x5x2mm) on the facial surfaces  ΔE_00_=1.9±1.0 | Omnichroma  (Tokuyama Dental Corporation, Tokyo, Japan)  N=40 cavities (3x5x2mm) on the facial surfaces  ΔE_00_=2.3±0.9 | Spectro ShadeTM Micro  (MHT Optic Research, Milan, Italy) | a: best match  b: intermediate match  c: poorest match | Distilled water | T_0_: 24h | Instrumental evaluation: ΔE values of Clearfil Majesty Esthetic samples were not statistically significantly lower than those of Omnichroma samples (p>0.05).  Visual evaluation: none of the samples showed poor match. |
| *Diab et al. 2023* | N=20 extracted human premolars | A3 | Filtek Z350 XT  (3M ESPE; St. Paul, MN, USA)  N=10 V class cavities (6x2x1.5mm) on the cervical third  ΔE_ab_=not specified | Omnichroma  (Tokuyama Dental Corporation, Tokyo, Japan)  N=10 V class cavities (6x2x1.5mm) on the cervical third  ΔE_ab_=not specified | VITA Easyshade 4.0 (VITA Zahnfabrik, Bäd Säckingen, Germany) | N/A | Distilled water | T_0_: 24h | No data at T_0_. |
| *Furusawa et al. 2023* | N=25 extracted human central incisors | A2 | Estelite Universal Flow  (Tokuyama Dental Corporation, Tokyo, Japan)  N=25 cilindrcal cavities (4x2mm) on the buccal side  ΔE_00_=not specified  Filtek Supreme Ultra Flow  (3M ESPE; St. Paul, MN, USA)  N=25 cilindrcal cavities (4x2mm) on the buccal side  ΔE_00_=not specified | Omnichroma  (Tokuyama Dental Corporation, Tokyo, Japan)  N=25 cilindrcal cavities (4x2mm) on the buccal side  ΔE_00_=not specified  Omnichroma flow  (Tokuyama Dental Corporation, Tokyo, Japan)  N=25 cilindrcal cavities (4x2mm) on the buccal side  ΔE_00_=not specified  EXP101 (Tokuyama Dental Corporation, Tokyo, Japan)  N=25 cilindrcal cavities (4x2mm) on the buccal side  ΔE_00_=not specified  EXP102  (Tokuyama Dental Corporation, Tokyo, Japan)  N=25 cilindrcal cavities (4x2mm) on the buccal side  ΔE_00_=not specified | CIE XYZ digital camera (RC500, PaPaLaB, Shizuoka, Japan) | N/A | 100% relative humidity at 37°C | T_0_: 24h | Instrumental evaluation: The lowest ΔE_00_ was measured with Omnichroma flow, but it was not significantly different from the others (p>0.05). Omnichroma exhibited significantly lower E_00_ adjustment than others (p<0.05). When compared with the conventional composites, Omnichroma flow outperformed both Estelite U flow and Filtek S flow (p<0.05). Omnichroma performed significantly better than Filtek S flow (p<0.05), but the ΔE_00_ of Omnichroma was similar to that of Estelite U flow (p>0.05). |
| *Gamal et al. 2022* | N=30 extracted human premolars | N/A | Fiber-reinforced composite  N=30 V class cavities (5x3x1.5mm) on the palatal side | Omnichroma  (Tokuyama Dental Corporation, Tokyo, Japan)  N=30 V class cavities (5x3x1.5mm) on the buccal side | Vita Easy shade compact  (Vita Zahnfabrik, Bad Sackingen, Germany | N/A | Distilled water | T_0_: 72h | No data at T_0_. |
| *Khayat 2024* | N=48 extracted human maxillary premolars | B2, A3, or A3.5 (n=16) | Filtek Z350  (3M ESPE; St. Paul, MN, USA)  N=48 round-shaped cavities (3x1.5mm) on the buccal or palatal side  ΔE_ab_=5.3±1.8 | Omnichroma  (Tokuyama Dental Corporation, Tokyo, Japan)  N=48 round-shaped cavities (3x1.5mm) on the buccal or palatal side  ΔE_ab_=7.4±1.8 | VITA Easyshade Advanced 4.0 (VITA Zahnfabrik, Bäd Säckingen, Germany) | 1=totally unacceptable  2=hardly acceptable  3=acceptable  4=small difference  5=no color difference | Distilled water | T_0_: 24h | Instrumental evaluation: The mean ΔE showed statistically significantly higher values for Omichroma than Filtek . The subgroup A3.5-Omnichroma (which recorded the highest mean ΔE among all subgroups) had significantly less color matching than the B2-Omnichroma and A3-Omnichroma subgroups.  Visual evaluation:  The visual scores of all Omnichroma and Filtek restorations ranged between 4 and 5. The mean VS values of Omnichroma restorations were comparable to the mean values of Filtek restorations in groups B2 and A3 (no statistically significant difference was detected between the types of restorations), but the difference in color matching was statistically significant between Omnichroma and Filtek restorations in group A3.5, revealing better visual color match for Filtek in A3.5 group. |
| *Kobayashi et al. 2021* | N=30 extracted human central incisors | A2 | Estelite Σ Quick  (Tokuyama Dental Corporation, Tokyo, Japan)  N=30 cylindrical cavities (4x2mm) on the labial side  ΔE_00_=not specified  Clearfil AP-X  (Kuraray Noritake, Tokyo, Japan)  N=30 cylindrical cavities (4x2mm) on the labial side  ΔE_00_=not specified | Omnichroma  (Tokuyama Dental Corporation, Tokyo, Japan)  N=30 cylindrical cavities (4x2mm) on the labial side  ΔE_00_=not specified | CIE XYZ digital camera (RC500, PaPaLaB, Shizuoka, Japan) | N/A | 100% relative humidity at 37°C | T_0_: 24h | Instrumental evaluation: Omnichroma exhibited the significantly lowest ∆E_00_ value (p<0.05), followed by Estelite Quick and Clearfil AP-X. |
| *Koi et al. 2024* | N=20 extracted human teeth | Light-tooth shade group: B1, A1, B2, D2, A2, C1, C2, and D4 (n=10)  Dark-tooth shade group: A3, D3, B3, A3.5, B4, C3, A4, and C4  (n=10) | Tetric EvoCeram (Ivoclar Vivadent, Schaan, Liechtenstein)  N=30 oval-shaped cavities (5x2mm) on the buccal side  ΔE_00_=2.6±1.3  Filtek Universal  (3M ESPE; St. Paul, MN, USA)  N=30 oval-shaped cavities (5x2mm) on the buccal side  ΔE_00_=2.8±1.1 | Omnichroma  (Tokuyama Dental Corporation, Tokyo, Japan)  N=30 oval-shaped cavities (5x2mm) on the buccal side  ΔE_00_=2.9±1.3  Admira Fusion x-tra (Voco, GmbH, Cuxhaven, Germany)  N=30 oval-shaped cavities (5x2mm) on the buccal side  ΔE_00_=4.0±1.8  Essentia Universal  (GC Corp., Tokyo, Japan)  N=30 oval-shaped cavities (5x2mm) on the buccal side  ΔE_00_=3.5±1.6 | CrystalEye  (Olympus, Tokyo, Japan) | N/A | Distilled water | T_0_: 24h | Instrumental evaluation:  For eight of the ten comparisons, the ΔE00 values were within 1.8 to 3.6, suggesting a mismatch that was moderately unacceptable. In two cases, the ΔE00 exceeded 3.6, suggesting an unacceptable mismatch, with both being for the Dark Shade Group, specifically Admira Fusion x-tra (5.17 ± 2.07) and Essentia Universal (3.74 ± 1.83).  There were no composites that achieved excellent or acceptable match.  A significant color difference between Light Shade Group and Dark Shade Group in Omnichroma and Admira Fusion x-tra. Omnichroma and Admira Fusion x-tra showed better color- matching ability in Light Shade Group than in Dark Shade Group. In Light Shade Group, Omnichroma showed better color-matching ability than Essentia Universal. In Dark Shade Group, Admira Fusion x-tra ΔE00 values were significantly higher than Omnichroma and Essentia Universal. |
| *Rosa et al. 2024* | N=10 extracted human third molars | A2, A3 | Filtek Universal  (3M ESPE; St. Paul, MN, USA)  N=5 cavities (6x2mm)  ΔE_ab_=10.8±4.3  ΔE_00_=6.2±2.3 | Omnichroma  (Tokuyama Dental Corporation, Tokyo, Japan)  N=5 cavities (6x2mm)  ΔE_ab_=12.5±3.9  ΔE_00_=7.2±2.1  Vittra APS Unique (FGM, Joinville, Brazil)  N=5 cavities (6x2mm)  ΔE_ab_=19.9±4.7  ΔE_00_=11.6±2.1  Charisma Diamond One  (Heraeus Kulzer, Hanau, Germany)  N=5 cavities (6x2mm)  ΔE_ab_=17.1±3.2  ΔE_00_=9.3±1.8 | Easyshade Compact Advance 5.0  (Vita-Zahnfabrik,  Bad Säckingen, Germany) | 0: perfect match/no difference in color  1: close match/small difference  2: good match/acceptable  3: poor match/hardly acceptable  4: mismatch/not acceptable | Water | T_0_: 1 week | Instrumental evaluation:  The best color match was observed for Filtek Universal followed by Omnichroma, Charisma Diamond One, and Vittra APS Unique.  Visual evaluation:  The highest scores were  observed for the Filtek Universal, with statistical differences from Omnichroma and Vittra Unique. |
| *Sanad et al. 2022* | N=90 extracted human anterior teeth | A2, A3, or A3.5 (n=30) | Filtek Z250  (3M ESPE; St. Paul, MN, USA)  N=30 V class cavities N=15 (3x0.5mm), N=15 (3x1.5mm)  ΔE_ab_=3.3±0.5  Ceram X SphereTEC  (Dentsply Sirona, Germany)  N=30 V class cavities N=15 (3x0.5mm), N=15 (3x1.5mm)  ΔE_ab_=5.4±0.7 | Omnichroma  (Tokuyama Dental Corporation, Tokyo, Japan)  N=30 V class cavities N=15 (3x0.5mm), N=15 (3x1.5mm)  ΔE_ab_=5.8±0.7 | VITA Easyshade V  (VITA Zahnfabrik, Bad Sackingen, Germany) | N/A | N/A | T_0_: before restoration  T_1_: after restoration | Instrumental evaluation:  Filtek™ Z250 XT showed the best results compared to the other two materials. |
| *Shaalan & El Rashidy 2023* | N=40 extracted human premolars | A2, A3 (n=10) | Filtek Z350 XT  (3M ESPE; St. Paul, MN, USA)  N=20 V class cavities (5x2mm)  ΔE_ab_=2.2±0.4  ΔE_00_=1.6±0.3 | Omnichroma  (Tokuyama Dental Corporation, Tokyo, Japan)  N=20 V class cavities (5x2mm)  ΔE_ab_=4.1±0.4  ΔE_00_=2.9±0.3 | VITA Easy shade V (VITA Zahnfabrik, Bäd Säckingen, Germany) | N/A | Distilled water | T_0_: after polymerization  T_1_:24h  T_2_:12 days | Instrumental evaluation: Multi-shade composite showed superior shade matching ability as compared to the single-shade one. |
| *Yamashita et al. 2023* | N=30 extracted human central incisors | A2 | Filtek Supreme Ultra  (3M ESPE; St. Paul, MN, USA)  N=30  ΔE_00_=not specified  Estelite Universal Flow  (Tokuyama Dental Corporation, Tokyo, Japan)  N=30  ΔE_00_=not specified | Omnichroma  (Tokuyama Dental Corporation, Tokyo, Japan)  N=30  ΔE_00_=not specified  Omnichroma flow  (Tokuyama Dental Corporation, Tokyo, Japan)  N=30  ΔE_00_=not specified  Clearfil Majesty ES Flow Universal  (Kuraray Noritake, Tokyo, Japan)  N=30  ΔE_00_=not specified  Clearfil Majesty ES Flow Universal dark  (Kuraray Noritake, Tokyo, Japan)  N=30  ΔE_00_=not specified | CIE XYZ digital camera (RC500, PaPaLaB, Shizuoka, Japan) | N/A | N/A | N/A | Instrumental evaluation: the universal-shade composites exhibited significantly better adjustment in E_00_, C* and h* than the conventional- shade composites regardless of thickness (p<0.05). |

**Supplementary Table 3.** Characteristics of the clinical trials that were a part of qualitative analysis.

| **STUDY** | **TYPE & NUMBER OF CAVITIES** | **TOOTH SHADES** | **MULTI-SHADE COMPOSITE & ΔE±DS** | **SINGLE-SHADE COMPOSITE & ΔE±DS** | **COLOR MEASUREMENT DIVICE** | **VISUAL SCALE** | **FOLLOW-UP** | **MAIN RESULTS** |
| --- | --- | --- | --- | --- | --- | --- | --- | --- |
| *Anwar et al. 2024* | N=40  2 molars or premolars with occlusal carious lesion in each patient | N/A | Tetric N-Ceram (Ivoclar Vivadent, Schaan, Liechtenstein)  N=20 | Omnichroma  (Tokuyama Dental Corporation, Tokyo, Japan)  N=20 | N/A | USPHS criteria for a color match and color stability as follows:  Alpha: The restoration closely resembles the translucency and color of neighboring dental tissues.  Bravo: The color and translucency of the restoration show a minor deviation from adjacent dental tissues, but it falls among the typical range of tooth shades.  Charlie: The restoration exhibits a significant discrepancy in shade and translucency when compared to the adjacent teeth structure, and the difference exceeds the typical range of tooth shade and translucency.  Marginal discoloration:  Alpha: No marginal discoloration observed,  Bravo: Marginal discoloration was present but limited in scope and did not extend significantly.  Charlie: Noticeable marginal discoloration, penetrating towards the pulp chamber.  The Alpha + Bravo score percentage was considered a clinical success. | Baseline, 1, 3, 6, 9, 12 months | Visual evaluation:  At the baseline, all restorations in the two groups revealed Alpha scores. After one, three, and six months, no statistically significant difference between both groups has been noticed. After nine and twelve months, the Tetric N group displayed a statistically significantly higher prevalence of Alpha scores and a lower prevalence of Bravo scores than the Omnichroma group. |
| *Favoreto et al. 2024* | N=120  2 non-carious cervical lesions in each patient | N/A | Vittra APS  (FGM, Joinville, Santa Catarina, Brazil)  N=60  ΔE_ab_=7.95±4.45  ΔE_00_=5.95±3.65 | Vittra APS Unique  (FGM, Joinville, Santa Catarina, Brazil)  N=60  ΔE_ab_=7.3±4.9  ΔE_00_=5.4±3.8 | VITA Easyshade  (VITA Zahnfabrik, Bad Säckingen, Germany) | 1: color stability and translucency  2: surface gloss/luster  3: surface color  4: anatomical form (aesthetic properties)  5: fracture and retention  6: marginal adaptation  7: marginal discoloration  8: patient perception (functional properties)  9: postoperative hypersensitivity  10: tooth vitality  11: recurrent of initial pathology  12: tooth integrity (enamel cracks)  13: periodontal response (always compared to the reference tooth) (biological properties) | Baseline, 1 week, 6, 12, 18 months | Instrumental evaluation: The single-shade resin composite used achieve the same color match when compared to a multi-shade resin composite after a period of 7 days in NCCLs.  Visual evaluation:  The restorations scored clinically very good (FDI) at baseline and after 18 months for all outcomes. |
| *Miranda et al. 2024* | N=120  2 non-carious cervical lesions in each patient | Distribution of the resin colors used to restore NCCLs with Admira Fusion: non-carious cervical lesions classified as 3rd- and 4th-degree sclerotic dentin were typically restored using A3O (38%) or A2O (12%) as the first layer, followed by a final layer of A3.5 or A3. On the other hand, lesions classified as 1st and 2nd degree of sclerotic dentin were restored using A2 (20%), A3.5 (20%), and A3 (10%) as the first layer, followed by either A3 or A2 as the final layer | Admira Fusion  (Voco, GmbH, Cuxhaven, Germany)  N=60  ΔE_00_=6.4±3.9 | Admira Fusion x-tra (Voco, GmbH, Cuxhaven, Germany)  N=60  ΔE_00_=6.3±3.9 | VITA Easyshade  (VITA Zahnfabrik, Bad Säckingen, Germany) | 1: color stability and translucency  2: surface gloss/luster  3: surface color  4: anatomical form (aesthetic properties)  5: fracture and retention  6: marginal adaptation/discoloration  7: patient perception (functional properties)  8: postoperative hypersensitivity  9: tooth vitality  10: recurrent of initial pathology  11: tooth integrity (enamel cracks)  12: periodontal response (always compared to the reference tooth) (biological properties) | T_0_: before restoration  T_1_: 1 week after restoration | Instrumental evaluation: No significant difference was observed when Admira Fusion was compared to Admira Fusion x-tra.  Visual evaluation: All restorations received the score of clinically very good at baseline and after 7 days. |
| *Porwal et al. 2024* | N=72  At least 2 molars or premolars with occlusal carious lesion in each patient. | N/A | Filtek Z350  (3M ESPE; St. Paul, MN, USA)  N=36  ΔE_00_=5.2±1.2 | Omnichroma Blocker and  Omnichroma  (Tokuyama Dental Corporation, Tokyo, Japan)  N=36  ΔE_00_=5.6±1.2 | DSLR Camera (Canon 13D)  Images were transferred to a digital adobe photoshop software  (adobe Inc.) for color matching analysis using CIELab coordinates;  L*, a*, b* coordinates were taken from the surface of the restoration and from intact tooth surface - 1 mm away from the  margin of the restoration | USPHS criteria,  encompassing various aspects such as marginal discolouration,  marginal adaptation, secondary caries, surface texture, colour  match, anatomic form, retention, and post-operative sensitivity. | Baseline, 6 months,  and 1 year | Instrumental evaluation:  no significant difference was observed in the ΔE  values between Fitlek and Omnichroma groups.  Visual evaluation:  Intergroup analysis showed that there was no statistically significant  difference between the two groups for all the variables of modified  USPHS. |
